# Clinical characteristics and prognosis of steroid-resistant nephrotic syndrome in children: a multi-center retrospective study

**DOI:** 10.1186/s13052-024-01817-4

**Published:** 2024-11-13

**Authors:** Sheng Li, Chao He, Yu Sun, Jie Chen, Yunguang Liu, Zengpo Huang, Weifang Huang, Yongqiu Meng, Wenjing Liu, Xianqiang Lei, Rihong Zhao, Zihui Lin, Chunlin Huang, Fengying Lei, Yuanhan Qin

**Affiliations:** 1 Department of Pediatrics, The First Hospital of Guangxi Medical University, Nanning, China; 2 Department of Pediatrics, The First Affiliated Hospital of University of South China, Hengyang, China; 3 Department of Pediatrics, Affiliated Hospital of Youjiang Medical College for Nationalities, Baise, Guangxi Province China; 4 Department of Pediatrics, Guigang people’s Hospital, Guigang, China; 5 Department of Pediatrics, Liuzhou Maternity and Child Healthcare hospital, Liuzhou, China; 6 Department of Pediatrics, Affiliated Hospital of Guilin Medical University, Guilin, China; 7 Department of Pediatrics, Maternity and Child Healthcare of Guangxi zhuang autonomous region, Nanning, China

**Keywords:** Steroid-resistant nephrotic syndrome, Clinical manifestation, Prognosis, Children

## Abstract

**Background:**

This study investigated the factors influencing the prognosis of children with steroid-resistant nephrotic syndrome (SRNS) in patients from the Guangxi region.

**Methods:**

We retrospectively analyzed clinical and pathological data of 279 patients with SRNS from six tertiary hospitals in Guangxi. Clinical data were compared between initial (I-SRNS) and secondary (S-SRNS) steroid resistance subgroups and Cox regression analysis was used to determine risk factors for chronic kidney disease (CKD) and CKD stage 5 (CKD5) in patients with SRNS.

**Results:**

The median age of onset was 54 months. Thirty-three patients had extra-kidney manifestations. Fifty-two, 24, 57, 33, and 41 patients had hypertension, acute kidney injury, vitamin D deficiency, high intraocular pressure, and dwarfism, respectively. One hundred eighty-two and 92 patients had I-SRNS and S-SRNS, respectively. There were significant differences in sex, ethnicity, family history, incidence of hematuria, clinical classification, efficacy of immune agents, and prognosis between groups (*P* < 0.05). Among the 279 cases of SRNS, 239 had normal kidney function, 37 developed CKD, and 16 had CKD5. An increase in serum creatinine level (HR = 1.003) was significantly associated with CKD in children with SRNS, and effective immunosuppressant therapy decreased the CKD risk (HR = 0.168). Patients with increased serum creatinine levels (HR = 1.003) and acute kidney injury (HR = 4.829) were more likely to progress to CKD5.

**Conclusions:**

Children with S-SRNS showed a higher response to immunosuppressants than those with I-SRNS. Effective immunosuppressant therapy was found to protect against CKD, whereas increased acute kidney injury was an independent risk factor for CKD5.

**Supplementary Information:**

The online version contains supplementary material available at 10.1186/s13052-024-01817-4.

## Introduction

Idiopathic nephrotic syndrome (INS) is one of the most common glomerular diseases in children, with an annual incidence of approximately 2–6.5 per 100,000 children, and is associated with ethnic and geographical factors [1]. The long-term prognosis of children with steroid-sensitive nephrotic syndrome is good, but 10–15% of patients with INS are steroid-resistant [2]. Most patients with steroid-resistant nephrotic syndrome (SRNS) need to be hospitalized repeatedly and require one or more immunosuppressants. The prognosis of these patients is poor, with nearly 20% developing stage 5 chronic kidney disease (CKD5) within 5–10 years [3]. MCD and FSGS are the most common histopathological features of SRNS. Although pathology findings show that the prognosis of MCD is good, there is some overlap between MCD and FSGS. Therefore, a single pathological diagnosis cannot comprehensively assess the condition and prognosis in children with SRNS. Compared to the pathological types, steroid response classification is more relevant for the prognosis of children with SRNS [4].

The pathogenesis of INS remains unclear. Heredity, genetic mutations, renal pathological changes, and abnormal glucocorticoid receptor signaling pathways may lead to steroid resistance. With the use of immunosuppressants, including mycophenolate mofetil, azathioprine, cyclosporine, or tacrolimus and the choice of biological agents, 50–70% of children with SRNS achieve some extent of clinical remission; some children still have multiple drug resistance. At the same time, long-term or repeated use of steroids can lead to side effects such as growth inhibition, hypertension, calcium deficiency, high intraocular pressure, and obesity. Therefore, the diagnosis and treatment of children with SRNS remain challenging.

There is a paucity of reports on independent predictors of prognosis of children with SRNS. This study analyzed the clinical data of children with SRNS in the Department of Nephrology in six tertiary hospitals in Guangxi, China, and aimed to explore the risk factors related to the prognosis of SRNS and to provide some reference for its evaluation.

## Materials and methods

### Patients

This multicenter retrospective study was conducted at the pediatric nephrology departments of six tertiary hospitals in Guangxi from January 2017 to October 2023. Initial SRNS (I-SRNS) was defined as a lack of remission after the first 4–6 weeks of steroid-inductive therapy, and secondary SRNS (S-SRNS) was defined as a lack of remission within 4 weeks after relapse in patients with previous steroid sensitivity [5].

Efficacy was determined as follows: (1) complete remission: urinary protein/creatinine (UP/CR) or 24-hour urine sample ≤ 20 mg/mmol or three or more negative urine dipstick tests; (2) partial remission: UP/CR > 20 to < 200 mg/mmol, serum albumin ≥ 30 g/L; and (3) response to immunosuppressants: if complete or partial remission was not achieved after receiving adequate calcineurin inhibitors (CNIs) and maintaining effective blood concentration for at least 6 months, CNI-resistant nephrotic syndrome was diagnosed. After 12 months of treatment with two different therapeutic doses of non-steroidal drugs, patients with SRNS who did not achieve complete remission developed multidrug-resistant nephrotic syndrome [2].

The estimated glomerular filtration rate (eGFR) was determined using the Schwartz formula [6]. Chronic kidney disease (CKD) was defined as abnormalities in kidney structure or function present for > 3 months, with staging based on a previously published CKD classification [7].

Hypertension was defined as blood pressure exceeding the 95th percentile of systolic or diastolic blood pressure for age, sex, and height [8]. Elevated intraocular pressure (IOP) in children is caused by the use of corticosteroids in the eye or whole body; when the IOP is more than 21 mmHg or the difference between the two eyes is more than 5 mmHg, ocular hypertension is diagnosed [9]. Acute kidney injury (AKI) was defined as a sudden decrease in kidney function within 48 h, with an absolute value of elevated serum creatinine ≥ 0.3 mg/dL at least twice, or a 50% increase in serum creatinine compared with the previous measurement, or a urine volume of less than 0.5 ml/(kg.h) lasting for more than 6 h [10]. According to professional organizations, a 25-dihydroxyvitamin D level of less than 20 ng/mL indicates vitamin D deficiency [11]. Dwarfism was defined as a height lower than the average of healthy individuals of the same sex, age, and race by two standard deviations [12].

The inclusion criteria were as follows: (1) age of onset of less than 18 years and (2) fulfilling the 2021 evidence-based guidelines for the diagnosis and treatment of SRNS [5]. Exclusion criteria were (1) children with secondary nephrotic syndrome; (2) incomplete clinical data.

### Clinical data collection and follow-up

The following data were collected: (1) general data: age, sex, nationality, height, family history (history of kidney disease in relatives within three generations), blood pressure, and extra-kidney clinical manifestations; (2) laboratory indicators: serum creatinine, eGFR, urinary sediment, 24-h urine protein or UP/CR, and kidney pathology; (3) clinical efficacy: the time when the urine protein testing turned negative and the efficacy of immunosuppressants; and (4) complications: hypertension, high IOP, dwarfism, and AKI. The study endpoint was October 31, 2023, CKD5 or death, as determined using clinical records and telephone tracing.

### Genetic analysis

Whole-exome sequencing was performed at MyGenostics Gene Technologies (Beijing, China) using genomic deoxyribonucleic acid isolated from the whole blood of the patient and their family members.

### Statistical analysis

Continuous variables were presented as medians and interquartile ranges (IQR), and categorical variables were presented as counts and percentages. Categorical variables were analyzed using the chi-squared or Fisher’s exact tests. The Mann–Whitney U test was used to compare the median differences in continuous variables between the two groups.

Univariate and multivariate Cox regression analyses were performed to calculate hazard ratios (HR) and 95% confidence intervals (CI) for CKD and CKD5. Statistical significance was set at a two-tailed *P* < 0.05. All statistical analyses and diagrams were performed and designed using R software version 4.3.1 (R Foundation for Statistical Computing, Vienna, Austria).

## Results

### Baseline characteristics

In this study, 279 patients with SRNS (male: female = 196:83) were enrolled. The median age at onset was 54 months (IQR 33, 94). Thirty-two patients (11.5%) had a relevant family history and 33 (11.8%) had extra-kidney manifestations, with neuropsychiatric symptoms being the most common, followed by cardiovascular symptoms (Table S1). Kidney biopsy was performed in 167 patients (60.6%). MCD (44.3%) and FSGS (38.3%) were the most common pathological types. There were 182 (65.2%) I-SRNS and 92 (33.0%) S-SRNS cases. Two hundred and fifty-two (90.3%) patients were treated with immunosuppressants and 171 (61.3%) showed complete or partial remission. Complications such as hypertension, AKI, vitamin D deficiency, high IOP, and dwarfism occurred in 52 (18.6%), 24 (8.6%), 57 (20.4%), 33 (11.8%), and 41 patients (14.7%), respectively. Among the 279 patients followed up for a median time of 54 months (IQR 30, 81), 37 (13.3%) progressed to CKD, including 21 (7.5%) with CKD2-4 and 16 (5.7%) with CKD5. Two patients with CKD5 died of severe infection and cardiac arrest respectively. Two patients were lost during follow-up (Table 1).

**Table 1:** Clinical characteristics and prognosis of 279 children with SRNS

Variables		Variables	
Age of onset (month), Median (Q1,Q3)	54.00(33.00, 94.00)	Steroid resistant-style, n (%)	
Sex, n (%)		I-SRNS	182 (65.2)
Male	196(70.3)	S-SRNS	92(33.0)
Female	83(29.7)	Uncertain	5(1.8)
Nation, n (%)		Response to immune therapy, n (%)	
Han	155(55.6)	Not use	27(9.7)
Zhuang	107(38.4)	Valid	171(61.3)
Yao	9(3.2)	Invalid	81(29.0)
Miao	3(1.1)	Complication	
Others	5(1.8)	Hypertension	52(18.6)
Family history, n (%)		AKI	24(8.6)
Yes	32(11.5)	Vitamin D deficiency	57(20.4)
No	247(88.5)	Ocular hypertension	33(11.8)
Extra-kidney manifestations, n (%)		Dwarfism	41(14.7)
Yes	33(11.8)	Prognosis, n (%)	
No	246(88.2)	Normal eGFR	239(85.7)
Kidney histology, n (%)		CKD2-4	21(7.5)
MCD	74(26.5)	CKD5	14(5.0)
FSGS	64(22.9)	Death	3(1.1)
MsPGN	19(6.8)	Lost follow-up	2(0.7)
MN	9(3.2)		
DMS	1(0.4)		
Follow-up time (month), Median (Q1,Q3)	54.00(30.0, 81.0)		

FSGS, focal segmental glomerulosclerosis; MCD, Minimal change disease; MsPGN, Mesangial proliferative glomerulonephritis; MN, membranous nephropathy; DMS, Diffuse Mesangial sclerosis; CKD, chronic kidney disease.

### Analysis of clinical characteristics and prognosis among different groups

#### Group of steroid resistance types

As shown in Table 2, there were 182 cases of I-SRNS and 92 cases of S-SRNS. The proportion of children with a family history of I-SRNS was higher than that of children with S-SRNS (13.7% vs. 5.4%, respectively, *P* = 0.019). In addition, there were significant differences in the ethnic distribution, incidence of hematuria, clinical classification, response to immune agents, and prognosis between the two groups (*P* < 0.05). There were no significant differences in the age of onset, incidence of complications, extra-renal manifestations, 24-h urinary protein level, serum creatinine level, and eGFR.

**Table 2:** Clinical characteristics of patients with initial and secondary steroid resistance

Variables	I-SRNS (*n* = 182)	S-SRNS (*n* = 92)	*P*-value
Age of onset (month), Median (Q1, Q3)	59.00 (29.25, 98.75)	53.00 (38.75, 84.00)	0.841
Sex, n(%)			**0.030**
Male	126 (69.2)	69 (75.0)	
Female	56 (30.8)	23 (25.0)	
Nation, n (%)			**0.043**
Han	108 (59.3)	42 (45.7)	
Zhuang	60 (33.0)	47 (51.1)	
Others	14 (7.2)	3 (3.2)	
Family history, n (%)	25 (13.7)	5 (5.4)	**0.019**
Extra-kidney manifestations, n (%)	23 (12.6)	8 (8.7)	0.103
24-hour urinary protein (mg), Median (Q1, Q3)	2559.3 (1549.1, 3761.6)	2588.05 (1832.7, 4181.9)	0.201
Scr (µmol/l), Median (Q1, Q3)	38.0 (25.0, 52.0)	37.00 (27.75, 52.00)	0.720
eGFR, Median (Q1, Q3)	119.33 (88.1, 151.8)	116.24 (98.7, 141.9)	0.478
Hematuria, n (%)	83 (45.6)	22 (23.9)	**< 0.001**
Response to immune therapy, n (%)			**< 0.001**
Valid	100 (54.9)	71 (77.2)	
Invalid	65 (35.7)	15 (16.3)	
Complication			
Hypertension, n (%)	37 (20.3)	13 (14.1)	0.147
AKI, n(%)	18 (9.9)	6 (6.5)	0.688
Vitamin D deficiency, n (%)	35 (19.2)	22 (23.9)	0.467
Ocular hypertension, n (%)	22 (12.1)	11 (12.0)	1.000
Dwarfism, n(%)	25 (13.7)	16 (17.4)	0.562
Follow-up time (month), Median (Q1, Q3)	47.00 (27.25, 73.00)	65.50 (45.75, 96.75)	**< 0.001**
Prognosis, n (%)			**0.034**
Normal eGFR	149 (81.9)	87 (94.6)	
CKD2-4	16 (8.8)	4 (4.3)	
CKD5	12 (6.6)	1 (1.1)	
Death	3 (1.6)	0 (0)	
Lost follow-up	2 (1.1)	0 (0)	

Scr, Serum creatinine; eGFR, estimated glomerular filtration rate.

There were 108 cases of I-SRNS and 57 cases of S-SRNS with kidney biopsy data, with no significant difference in the distribution of pathological types between the two groups (*P* = 0.183). The main pathological types were MCD and FSGS, followed by MsPGN (Table S2).

### Analysis of risk factors of progression to CKD in children with SRNS

Of the 279 children with SRNS, two hundred and thirty-nine (85.7%) had normal kidney function and 38 progressed to CKD. Univariate Cox regression analysis showed that age at onset (HR = 1.008, 95%CI: 1.001–1.016; *P* = 0.033), serum creatinine increase (HR = 1.002, 95%CI: 1.001–1.003; *P* = 0.004), FSGS (HR = 3.875, 95%CI: 1.535–9.786; *P* = 0.033), and AKI (HR = 2.201, 95%CI: 1.029–4.709; *P* = 0.042) were risk factors for CKD. However, the risk of CKD was significantly lower in patients with I-SRNS (HR = 0.169, 95%CI: 0.038–0.748; *P* = 0.019), S-SRNS (HR = 0.040, 95%CI: 0.007–0.218; *P* < 0.001), higher eGFR (HR = 0.992, 95%CI: 0.985–0.998; *P* = 0.012), and effective immunosuppressant therapy (HR = 0.127, 95%CI: 0.037–0.443; *P* = 0.001). Multivariate regression analysis revealed that an increase in serum creatinine level (HR = 1.003, 95%CI: 1.000-1.005; *P* = 0.033) was significantly associated with the development of CKD, whereas effective immunosuppressant therapy (HR = 0.168, 95%CI: 0.032–0.892; *P* = 0.036) was a protective factor against (Table 3).

**Table 3:** Risk factors for progression from SRNS to CKD

Variables	Univariate Cox regression		Multivariate Cox regression	
	HR (95%CI)	*P*-value	HR (95%CI)	*P*-value
Steroid resistant-style (reference, uncertain)				
I-SRNS	0.169 (0.038, 0.748)	**0.019**	2.017 (0.162, 25.152)	0.586
S-SRNS	0.040 (0.007, 0.218)	**< 0.001**	0.882 (0.058, 13.332)	0.928
Age of onset	1.008 (1.001, 1.016)	**0.033**	1.007 (0.997, 1.016)	0.167
Sex (reference, male)				
Female	1.046 (0.519, 2.108)	0.900		
Nation (reference, Han)				
Zhuang	0.630 (0.325, 1.220)	0.171		
Others	1.009 (0.135, 7.532)	0.993		
Family history (reference, no)				
Yes	6.880 (3.478, 13.607)	**< 0.001**	1.459 (0.464, 4.582)	0.518
Extra-kidney manifestations (reference, no)				
Yes	1.027 (0.364, 2.896)	0.960		
24-hour urinary protein	1.000 (1.000, 1.000)	0.787		
Scr	1.002 (1.001, 1.003)	**0.004**	1.003 (1.000, 1.005)	**0.033**
eGFR	0.992 (0.985, 0.998)	**0.012**	1.001 (0.996, 1.005)	0.768
Hypertension (reference, no)				
Yes	1.004 (0.487, 2.071)	0.991		
Kidney biopsy (reference, MCD)				
FSGS	2.366 (1.073, 5.216)	**0.033**	1.548 (0.490, 4.892)	0.780
MsPGN	1.141 (0.245, 5.302)	0.867		
MN	1.841 (0.230, 14.729)	0.565		
DMS	5.460 (0.686, 43.441)	0.109		
Hematuria (reference, no)				
Yes	2.575 (1.366, 4.853)	**0.003**	0.130 (0.005, 3.162)	0.210
Response to immune therapy (reference, no use)				
Valid	0.127 (0.037, 0.443)	**0.001**	0.168 (0.032, 0.892)	**0.036**
Invalid	1.351 (0.520, 3.511)	0.536		
AKI (reference, no)				
Yes	2.201 (1.029, 4.709)	**0.042**	1.866 (0.773, 4.504)	0.165

### Analysis of risk factors of progression to CKD5 in children with SRNS

In this study, 16 children with SRNS developed CKD5. Univariate regression analysis showed that family history (HR = 7.320, 95%CI: 2.582–20.756; *P* < 0.001), elevated serum creatinine (HR = 1.003, 95%CI: 1.001–1.004; *P* < 0.001), and AKI (HR = 4.419, 95%CI: 1.669–11.703; *P* = 0.003) were significantly associated with CKD5. The risk of developing CKD5 was lower in children with S-SRNS (HR = 0.011, 95%CI: 0.001–0.206; *P* = 0.003), increased eGFR (HR = 0.986, 95%CI: 0.976–0.996; *P* = 0.005), and effective immunosuppressant therapy (HR = 0.133, 95%CI: 0.018–0.96; *P* = 0.046). Multivariate regression analysis revealed that elevated serum creatinine (HR = 1.003, 95%CI: 1.000-1.007; *P* = 0.038) and AKI (HR = 4.829, 95%CI: 1.113–20.947; *P* = 0.035) were risk factors for progression to CKD5 (Table 4).

**Table 4:** Risk factors for the progression of SRNS to CKD5

Variables	Univariate Cox regression		Multivariate Cox regression	
	HR (95%CI)	*P*-value	HR (95%CI)	*P*-value
Steroid resistant-style (reference, uncertain)				
I-SRNS	0.123 (0.014, 1.069)	0.057		
S-SRNS	0.011 (0.001, 0.206)	**0.002**	0.125 (0.003, 5.966)	0.291
Age of onset	0.999 (0.987, 1.012)	0.899		
Sex (reference, male)				
Female	1.748 (0.674, 4.534)	0.251		
Nation (reference, Han)				
Zhuang	0.620 (0.234, 1.643)	0.336		
Others	0.000 (0.000, Inf )	0.998		
Family history (reference, no)				
Yes	7.320 (2.582, 20.756)	**< 0.001**	2.046 (0.344, 12.169)	0.431
Extra-kidney manifestations (reference, no)				
Yes	1.817 (0.525, 6.295)	0.346		
24-hour urinary protein	1.000 (0.999, 1.000)	0.124		
Scr	1.003 (1.001, 1.004)	**< 0.001**	1.003 (1.000, 1.007)	**0.038**
eGFR	0.986 (0.976, 0.996)	**0.005**	1.001 (0.996, 1.007)	0.624
Hypertension (reference, no)				
Yes	1.929 (0.728, 5.110)	0.186		
Kidney biopsy (reference, MCD)				
FSGS	4.205 (0.888, 19.919)	0.070		
MsPGN	5.196 (0.725, 37.233)	0.101		
MN	10.456 (0.905, 120.788)	0.060		
DMS	21.715 (1.929, 244.396)	0.013	0.209 (0.002, 28.620)	0.533
Hematuria (reference, no)				
Yes	2.243 (0.885, 5.682)	0.089		
Response to immune therapy (reference, no use)				
Valid	0.133 (0.018, 0.961)	**0.046**	0.320 (0.011, 9.095)	0.504
Invalid	1.575 (0.355, 6.996)	0.550		
AKI (reference, no)				
Yes	4.419 (1.669, 11.703)	**0.003**	4.829 (1.113, 20.947)	**0.035**

### Whole-exome sequencing results

Of the 279 children with SRNS, 89 underwent whole-exome sequencing (WES). Sixty-six (36.3%), 18 (22.0%) and 5 (100%) patients with I-SRNS, S-SRNS, and uncertain steroid resistance-type SRNS, respectively, employed WES (Table S3). The negative genetic testing rate of patients with S-SRNS was 100%. There was a significant difference among the three groups of children with different types of SRNS (*P* < 0.001). In 22 patients, 26 potentially pathogenic gene mutations associated with SRNS were identified, with these variants spanning across eight different genes (Figure S1).

## Discussion

In this study, we summarized and analyzed the clinical characteristics and prognosis of 279 children with SRNS. The median age at onset was 54 months and the male-to-female ratio was 2.36, which is consistent with other report [13]. Thirty-two and 33 children with SRNS had a family history of SRNS and extra-kidney manifestations, respectively. Of the 32 cases of SRNS with a family history, 10 patients progressed to CKD, and six of them progressed to CKD5. Multivariate Cox regression analysis showed that family history was not a risk factor for the progression to CKD and CKD5, which may be due to the short follow-up period. In addition, neuropsychiatric diseases were more common in the extra-renal phenotypes, which is consistent with previous reports [14].

Previous research have shown that FSGS and MCD are the most common pathological types in children with SRNS [15]. In this cohort, the pathological classifications of MCD and FSGS accounted for 26.5% and 22.9% of cases, respectively, which is similar to previous study [16]. In addition, a female patient with SRNS and pathological findings of DMS, whose onset age was 48 months, had a family history (her sister was diagnosed with INS at the age of 5), and absence of patella, fingernails, and right lower extremity. DMS is the most common pathology in children with SRNS and is associated with the WT1 [17], TRPC6 [18], NUP93 [19] gene variants. However, the WES result of this patients revealed a novo mutation of LMX1B, c.794 T-patellar G (p.Val265Gly), suggesting that genotypes and phenotypes may not necessarily be consistent. In this study, nine cases and 19 cases of SRNS with the FSGS and MCD pathological types progressed to CKD, respectively. Multivariate Cox regression analysis showed that there was no significant difference between patients with and without CKD or between the MCD and FSGS pathological types in the prognosis of SRNS. Previous studies have reported that MCD and FSGS may show different pathological changes during the development of the same disease and cannot be used as independent diagnoses [20]. Therefore, Zee et al. suggested that new feature descriptions should be added to the standardized clinicopathological reports describing MCD/FSGS histopathology, combined with clinical, environmental, genetic, or molecular biomarkers, to predict the progression of SRNS and the treatment response [21].

The proportion of patients with S-SRNS in this study was 33.0%, which is similar to previous report [22]. Although S-SRNS has been known for decades, its mechanism of action has not been fully clarified. Recent study have shown that it may be a high-risk factor for recurrence after transplantation [23]. Furthermore, our analysis reveals that the S-SRNS subgroup showed a more efficient remission rate for immunosuppressant efficacy, aligning with the findings of the most recent multi-center report [23]. Our study also discovered that effective immunosuppressive therapy is a protective factor to prevent children with SRNS from developing into CKD. This aligns with the guideline recommendation that CNIs can be employed as the initial therapy for children with SRNS [24].

INS is prone to recurrence and chronicity and requires long-term or repeated use of steroids and immunosuppressants. In addition to the increased risk of infection and venous thromboembolism, patients are prone to other complications. In our study, approximately 16.8% of patients had hypertension. While another report suggested that hypertension was observed in only 8.0% of patients with SRNS [25]. These discrepancies may be attributed to differences in the age at onset. It is worth noting that 8.6% of children with SRNS in our cohort experienced AKI, and Rheault et al. also found that SRNS was related to the risk of AKI [26]. A further analysis showed that AKI was an independent risk factor for the progression of CKD5 in children with SRNS, and the incidence of CKD5 was 4.829 times higher than that in children without AKI, which is consistent with a Japanese large-sample report [22]. This may be related to the use of nephrotoxic drugs in patients with SRNS, which aggravates the deterioration of kidney function. Vitamin D deficiency was the most common complication in this study, with an incidence rate of 20.4%. However, it was previously reported that 77.1% of children with INS have severe vitamin D deficiency [27]; therefore, the relationship between vitamin D deficiency and INS remains a concern. In addition to the fact that serum vitamin D content in children with INS is accompanied by the loss of vitamin D binding protein in urine. It has been suggested that glucocorticoid and vitamin D receptors have analogous molecular structures, long-term use of glucocorticoids can competitively inhibit the synthesis and utilization of vitamin D [28]. Moreover, 33 patients developed ocular hypertension, most of whom were controlled after regular treatment to lower intraocular pressure, and only one patient (3.0%) underwent surgical treatment. In this study, 14.7% of children were diagnosed with dwarfism. However, Lee et al. concluded that in childhood-onset INS, the augmentation in height is predominantly influenced by genetic target height, rather than complications caused by steroids or immunosuppressants, unless in cases of progression to CKD/CKD5 [29]. In this study, the median follow-up time was only 54 months, and no patients were followed up as adults; therefore, an accurate conclusion could not be drawn. In short, this study found that AKI is an independent risk factor for predicting the progression of SRNS to CKD5. Hypertension and ocular complications are common in children with SRNS; however, their prognoses are good.The diagnosis of dwarfism in children with SRNS requires a long evaluation period.

In this study, 89 patients with SRNS (31.9%) were subjected to WES analysis, and the positive rate of pathogenic gene mutations was 24.7%, which is roughly consistent with other study [30].

Our study has several limitations. First, this was a retrospective study of nearly 7 years, which inevitably has some bias. Second, the follow-up time was relatively short, and the accuracy of evaluating the complications and prognosis of SRNS was limited. Third, this study only summarized and analyzed the clinical characteristics of SRNS in Guangxi, and its generalizability is limited.

## Conclusions

Most children with SRNS in this region were male, neuropsychiatric symptoms were the most common extra-kidney manifestations, and MCD and FSGS were identified as the main pathological types of SRNS. Effective immunosuppressant therapy was found to be a protective factor against CKD in children with SRNS, whereas AKI was identified as an independent risk factor for CKD5.

## Electronic supplementary material

Below is the link to the electronic supplementary material.

Supplementary Material 1

Supplementary Material 2

Supplementary Material 3

Supplementary Material 4

## Data Availability

Datasets from this study are available from the corresponding author upon request.
