# Supplementary material for: Clinical characteristics and prognosis of steroid-resistant nephrotic syndrome in children: a multi-center retrospective study
Source: Ital J Pediatr. 2024 Nov 13;50:242. doi: 10.1186/s13052-024-01817-4 (PMC11559144; doi:10.1186/s13052-024-01817-4)
Supplement: Supplementary file 1 — Supplementary Material 1 [file 13052_2024_1817_MOESM1_ESM.docx]

Table S1. Detailed extra-renal manifestations in 33 children with SRNS

| Extra-kidney manifestations | N. | Mean age of onset (month) |
| --- | --- | --- |
| Neuropsychiatric |  |  |
| Psychomotor retardation | 11 | 68.5 |
| Epilepsy | 2 | 61.5 |
| Autism | 1 | 41.0 |
| Cardiovascular system |  |  |
| Patent foramen ovale | 3 | 61.3 |
| Atrial septal defect | 2 | 12.5 |
| Ventricular septal defect Digestive system | 1 | 80.0 |
| Diaphragmatic/esophageal hiatal hernia | 2 | 63.5 |
| Megacolon | 1 | 33.0 |
| Inguinal hernia | 2 | 35.0 |
| SNHL | 1 | 147.0 |
| Skin system | 3 | 84.3 |
| Skeletal muscle system | 3 | 58.3 |
| Others：adrenal gland space-occupying，visceral ectopia | 4 | 46.7 |

SNHL: Sensorineural Hearing Loss
