# Supplementary material for: Clinical characteristics and prognosis of steroid-resistant nephrotic syndrome in children: a multi-center retrospective study
Source: Ital J Pediatr. 2024 Nov 13;50:242. doi: 10.1186/s13052-024-01817-4 (PMC11559144; doi:10.1186/s13052-024-01817-4)
Supplement: Supplementary file 2 — Supplementary Material 2 [file 13052_2024_1817_MOESM2_ESM.docx]

Table S2. Pathological SRNS types in patients with initial and secondary steroid resistance

| Type of Pathological | N. (%) | I-SRNS (%) | S-SRNS (%) | *P-*value (Fisher) |
| --- | --- | --- | --- | --- |
| MCD | 74 (44.8) | 42 (38.9) | 32 (56.1) |  |
| FSGS | 62 (37.6) | 43 (39.8) | 19 (33.3) |  |
| MsPGN | 19 (11.5) | 14 (13.0) | 5 (8.8) | 0.183 |
| MN | 9 (5.5) | 8 (7.4) | 1 (1.8) |  |
| DMS | 1 (0.6) | 1 (0.9) | 0 (0) |  |
