# Supplementary material for: Clinical characteristics and prognosis of steroid-resistant nephrotic syndrome in children: a multi-center retrospective study
Source: Ital J Pediatr. 2024 Nov 13;50:242. doi: 10.1186/s13052-024-01817-4 (PMC11559144; doi:10.1186/s13052-024-01817-4)
Supplement: Supplementary file 3 — Supplementary Material 3 [file 13052_2024_1817_MOESM3_ESM.docx]

Table S3. Whole-exome sequencing findings in patients with initial and secondary steroid resistance

| Type of  SRNS | I-SRNS (%) | S-SRNS (%) | Uncertain (%) | *P*-value |
| --- | --- | --- | --- | --- |
| Not done Positive Negative | 116 (63.7)  18 (9.9)  48 (26.4) | 74 (80.4) 0 (0)  18 (19.6) | 0 (0)  4 (80.0%) 1 (20.0%) | ＜0.001 |
