# Supplementary figures and images for: Clinical characteristics and prognosis of steroid-resistant nephrotic syndrome in children: a multi-center retrospective study
Source: Ital J Pediatr. 2024 Nov 13;50:242. doi: 10.1186/s13052-024-01817-4 (PMC11559144; doi:10.1186/s13052-024-01817-4)

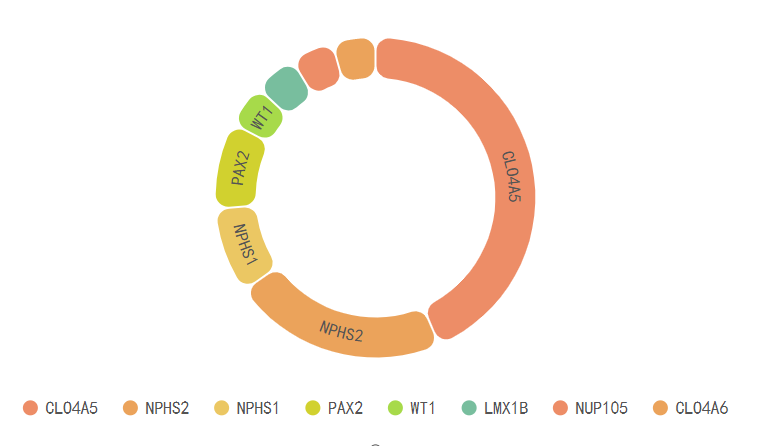


Fig.S1 Summary of identified genetic variants in the SRNS cohort.

Supplement: Supplementary file 4 — Supplementary Material 4 [file 13052_2024_1817_MOESM4_ESM.doc]
